# Vanadium Dioxide-Based Terahertz Metamaterial Devices Switchable between Transmission and Absorption

**DOI:** 10.3390/mi13050715

**Published:** 2022-04-30

**Authors:** Haoqing Jiang, Yue Wang, Zijian Cui, Xiaoju Zhang, Yongqiang Zhu, Kuang Zhang

**Affiliations:** 1 Key Laboratory of Ultrafast Photoelectric Technology and Terahertz Science in Shaanxi, Xi’an University of Technology, Xi’an 710048, China; 2200920071@stu.xaut.edu.cn (H.J.); 1200913002@stu.xaut.edu.cn (Z.C.); 1190911001@stu.xaut.edu.cn (X.Z.); 2190920061@stu.xaut.edu.cn (Y.Z.); 2 Foundation Department, Engineering University of PAP, Xi’an 710086, China; 3 School of Electronics and Information Engineering, Harbin Institute of Technology, Harbin 150001, China; zhangkuang@hit.edu.cn

**Keywords:** metamaterial, vanadium dioxide, tunable metamaterials, perfect absorption

## Abstract

Terahertz metamaterial plays a significant role in the development of imaging, sensing, and communications. The function of conventional terahertz metamaterials was fixed after fabrication. They can only achieve a single function and do not have adjustable characteristics, which greatly limits the scalability and practical application of metamaterial. Here, we propose a vanadium dioxide-based terahertz metamaterial device, which is switchable between being a transmitter and an absorber. The transmission and absorption characteristics and temperature tunable properties of phase change metamaterials in the terahertz band were investigated. As the temperature of vanadium dioxide is varied between 20 °C and 80 °C, the device can switch between transmission and quad-band resonance absorption at the terahertz frequency range, with a high transmission rate of over 80% and a peak absorbance of 98.3%, respectively. In addition, when the device acts as an absorber, the proposed metamaterial device is tunable, and the modulation amplitude can reach 94.3%; while the device is used as a transmissive device, the modulation amplitude of the transmission peak at 81%. The results indicate that the proposed metamaterial device can promote the applications of terahertz devices, such as switching, modulation, and sensing.

## 1. Introduction

Terahertz (THz) technology has recently attracted extensive attention due to its unique advantages. For example, THz waves have high penetration and very low energy. THz radiation plays a significant role in the development of sensors, biomedicine, radar, security detection, and imaging [1,2]. However, few natural materials can interact with THz radiation. Therefore, researchers are interested in a metamaterial that can respond to THz waves [3]. Metamaterials are artificially designed sub-wavelength electromagnetic structures with unique physical properties [4], which makes them a desirable solution to this problem. Metamaterials have fast growth in THz perfect absorbers, perfect lenses, and transmitters. Among them, THz perfect absorbers based on metamaterials have many advantages, such as ultra-thin dielectric layers, lightweight, and controllable performance (including frequency and absorbance) [5]. As a result, perfect absorbers, including single and double band, multi-band, and broadband devices, have become a hot topic for researchers [6,7,8]. However, the function of conventional THz metamaterials was fixed after their preparation. Therefore, the study of tunability and multi-functionality in metamaterials is essential for the practical application of THz waves.

To address this problem, researchers have proposed various solutions over the past few years, including the idea that metamaterial devices could be tunable over a range by changing geometry parameters and dielectric properties of metamaterials [9,10]. Furthermore, the intrinsic properties of tunable materials, such as conductivity, electron mobility, and dielectric constant [9,11], can be efficiently modulated by external excitations to influence the electromagnetic properties of functional devices. Recently, vanadium dioxide (VO_2_), graphene, black phosphorus, and liquid crystals were introduced into the metamaterial design to actively control the optical and electrical properties of the functional devices in the THz range [12,13,14]. Among them, VO_2_ is a phase-changing material that can switch between an insulator and conductor by external excitations (including photo-excitation, electrical bias, and thermal tuning) [15,16,17,18,19]. During the transition process, the conductivity of VO_2_ can be changed by up to five orders of magnitude [20], which suggests that it is an appropriate candidate for an active tuning device. However, the previously reported tunable devices only have a single function. Therefore, it is desirable to integrate diversified functionalities into a single device.

This study presents a VO_2_-based THz metamaterial device, which is switchable between being a transmitter and an absorber. The tunability of such devices is achieved by changing the temperature and therefore driving the phase change of the VO_2_ material. Four discrete resonant absorption bands are achieved in the THz region, two of which can achieve high absorption rates (>98%) for perfect absorption [21,22]. In addition, the device can switch between transmission (higher than 80% transmissivity) and multiband absorption (absorption can reach up to 98.3%) by changing the temperature. The absorption rate is tunable from 3.9% to 98.3%, and its modulation amplitude can reach up to 94.4%. The transmission peak of the transmission device can be dynamically adjusted, with temperature change over a range of 0.02% to 81.8%, showing a modification amplitude over 81%. This study provides a design method for a multi-band tunable dual-functional device in the THz band, which can also be used for more applications in other bands by varying the structural dimensions.

## 2. Materials and Methods

**Figure 1:**
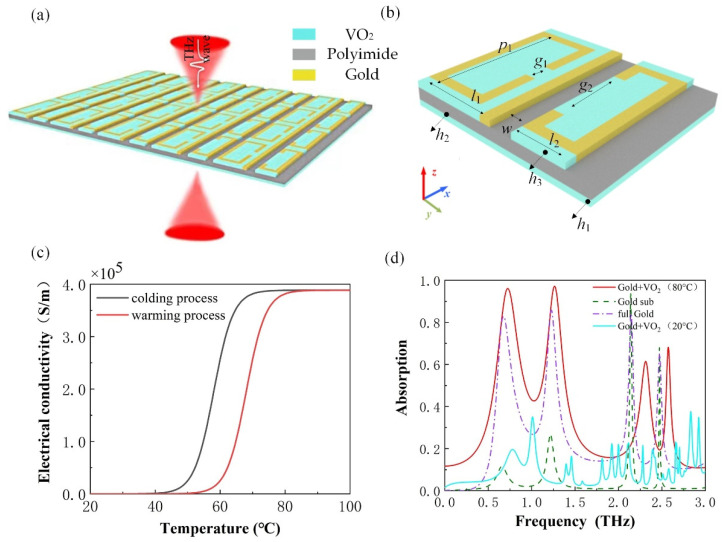
(**a**) Schematic diagram of the array and (**b**) unit multifunctional tunable functional device structure; (**c**) VO_2_ films conductivity as a function of temperature (warming process *T*_0_ = 68 °C, cooling process *T*_0_ = 58 °C); (**d**) Absorption response of the absorber for different material cases of metal and VO_2_.

The designed dual-function tunable device is exhibited in Figure 1a. As shown in Figure 1b, the fundamental unit of the device is composed of a 3-layer structure: the surface layer consists of VO_2_ and gold, the intermediate polyimide layer offers a transmission space to THz waves, and the bottom layer is made up of a thin film of VO_2_. The surface resonant layer of two square open split-ring resonators made of metallic material form the pattern, together with VO_2_. The metallic and VO_2_ layers and the VO_2_ substrates are *h*_1_ and *h*_3_, respectively. The thickness of the polyimide spacer is *h*_2_. Polyimide can be considered as a lossy dielectric with a relative permittivity of *ε* = 3.5 + 0.00945i. The conductivity of Au is σ_Au_ = 4.56 × 10^7^ S/m, the gold used here can be considered a lossy material, and the material properties are shown below (Table 1). The repeat period is *p*_1_, the width of the structural unit is *l*_1_ and *l*_2_, respectively, and the width of the inter-structural slit is *w*. The gap size of the split ring resonator is *g*_1_ and *g*_2_, respectively. In our simulation, *h*_1_ = 0.2 μm, *h*_2_ = 20 μm, *h*_3_ = 0.2 μm, *p*_1_ = 150 μm, *l*_1_ = 90 μm, *l*_1_ = 90 μm, *w* = 14 μm *g*_1_ = 35 μm, and *g*_2_ = 80 μm. Those values are constant unless otherwise specified.

CST Microwave Studio 2018 software (CST from Dassault Systemes, Framingham, MA, USA) has been used to investigate the electromagnetic responses of metamaterial devices. The adaptive tetrahedral mesh refinement was used for the mesh. In the simulation, the 𝑥- and 𝑦-directions were set as unit cell boundary conditions and the 𝑧-direction as open (add space) boundary conditions, which were used to construct an infinite arrangement of structures to match the periodic array. The optical properties of VO_2_ can be calculated with the Drude model [23,24], which is written by $\varepsilon \left(\omega \right)=\varepsilon_{\infty }-\frac{\omega_{p}^{2}\left(\sigma \right)}{\omega_{2}+i\gamma \omega }$, where $\varepsilon_{\infty }$ = 9, and γ = 5.75 × 10^13^ rad/s is the frequency of collision. The conductivity dependent plasma frequency ω(σ) at σ can be approximately described as $\omega_{p}^{2}\left(\sigma_{{\mathrm{vo}}_{2}}\right)=\frac{\sigma_{{\mathrm{vo}}_{2}}}{\sigma_{0}}\omega_{p}^{2}\left(\sigma_{0}\right)$, where $\sigma_{0}$ = 3 × 10^5^ S/m, ω_ω_ ($\sigma_{0}$) = 1.4 × 10^15^ rad/s. The parameters were set in the software according to the Drude model described in the manuscript. Previous studies have reported that the conductivity of VO_2_ can be increased from 200 S/m to 4 × 10^5^ S/m [25,26]. In this paper, the conductivity of VO_2_ needs to be analysed by effective medium theory (EMT).

During the phase transition process of VO_2_, the dielectric state of the VO_2_ film would be replaced by the metal state. In order to characterize VO_2_ materials in incomplete phase transitions, EMT is required. Currently, Maxwell–Garnett EMT and Bruggeman EMT are commonly used methods [27,28], but Maxwell–Garnett EMT is not suitable for phase transformation where the volume fraction of the metal component is greater than 20%, while the Bruggeman EMT can be utilized for VO_2_ thin films. The dielectric constant $\varepsilon_{c}$ can be expressed as:

(1) $$\varepsilon_{c}=\frac{1}{4}(\varepsilon_{D}(2-3f_{\left(T\right)}))+\varepsilon_{M}(3f_{\left(T\right)}-1)+\sqrt{\left(\varepsilon_{D}(2-3f_{\left(T\right)})+\varepsilon_{M}{\left(3f_{\left(T\right)}-1\right)}^{2}+8\varepsilon_{D}\varepsilon_{M}\right)}$$

where $\varepsilon_{D}$ and $\varepsilon_{M}$ are the dielectric functions of the VO_2_ thin films in the insulation and metal phase, respectively. The dielectric function of the metallic phase $\varepsilon_{M}$ is represented by the Drude model with the activity of the dielectric function of the insulator component $\varepsilon_{D}$ = 9. In addition, the volume fraction of the metal component *f*_(*T*)_ can be described as:

(2) $$f_{\left(T\right)}=f{\left(1-\frac{1}{1+exp(\frac{T-T_{0}}{\Delta T})}\right)}_{max}$$

where *T*_0_ is the phase transition temperature of VO_2_, ∆T is the transition width. Here, *T*_0_ = 68 °C and ∆T = 3 °C are obtained from the experiment [29]. By combining Equation (1) with (2), the conductivity of VO_2_ thin films corresponding to different temperatures is expressed as $\sigma_{VO_{2}}=-i\varepsilon_{0}\omega \left(\varepsilon_{c}-1\right)$. Moreover, the electrical conductivity can be varied as the temperature change in the VO_2_ thin films as shown in Figure 1c.

At a temperature of 80 °C, the electromagnetic response of the devices proposed in the study was first demonstrated with VO_2_ thin films in the metallic phase. Figure 1d shows the absorption of the device under different materials. The absorption can be calculated by $A\left(\omega \right)=1-R\left(\omega \right)-T\left(\omega \right)=1-\left|S_{11}\left(\omega \right){|}^{2}-\right|S_{21}\left(\omega \right){|}^{2}$, where *R*(ω) represents the reflection, and *T*(ω) is the transmission. The reflection *S*_11_(ω) and the transmission *S*_21_(*ω*) can be obtained by simulation. The device has four absorption peaks in the range of 0.1–3.0 THz, of which two absorption rates are higher than 98% at the resonant frequencies of about 0.66 THz and 1.22 THz, and the absorption efficiency is 98.2% and 98.3%, respectively.

If the bottom layer material is replaced from VO_2_ to gold, the absorption rate becomes 82.9% and the first absorption peak has a red-shift. The absorption rate becomes 85.7% for the second band and the peak frequency shifted to 1.23 THz. In this case, no peak values can exceed 90%. If the VO_2_ in the bottom and top layers is replaced with gold, the absorption rate of the first band becomes 11.5%, and the peak frequency moves to 0.66 THz. The absorption rate becomes 27.0% for the second band, and the peak frequency moves to 1.22 THz. In this case, only one peak was higher than 90%. The absorption peak is located at 2.14 THz with an absorption rate of over 93.8%. However, when using gold material, the absorption peak of this device is not adjustable and cannot achieve bi-functionality, which is not the desired goal of our study. Through the analysis above, the performances of VO_2_ material are better than that of gold in the proposed device.

## 3. Results and Discussion

The absorption response of the devices is shown in Figure 1d. Quad-band absorption resonance bands with narrow bandwidths at frequencies of *f*_1_ = 0.66 THz, *f*_2_ = 1.22 THz, *f*_3_ = 2.14 THz, and *f*_4_ = 2.48 THz were realized. Further structural optimization can provide the ability to achieve even more perfect absorption peaks. These results indicate that devices have good absorption modes. To illustrate the origin of the modes, the two dual-mode perfect absorptions achieved over 98% absorption at *f*_1_ and *f*_2_ are named mode 1 and mode 2, respectively.

**Figure 2:**
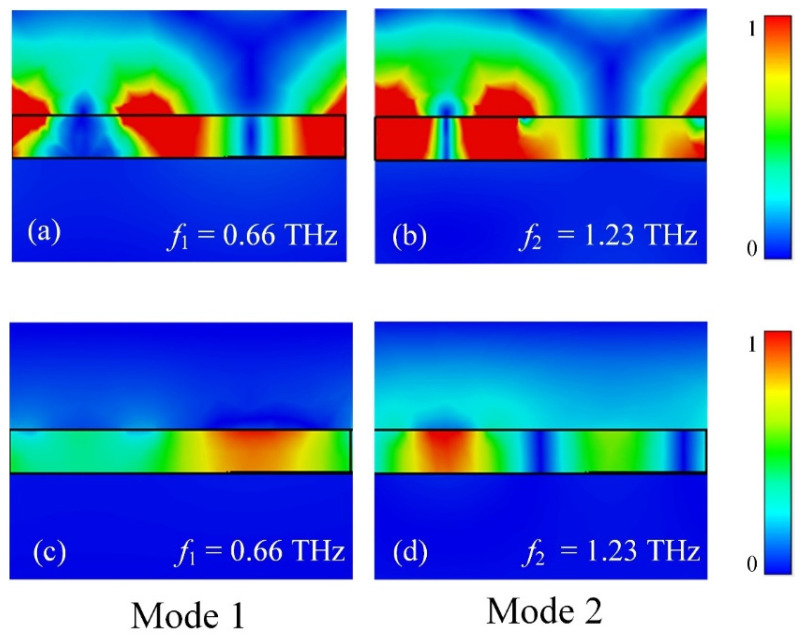
(**a**,**b**) The distribution of electric |*E*| field at the absorber’s resonance frequency. (**c**,**d**) The distribution of magnetic |*H*| field at the absorber’s resonance frequency.

The resonance mechanism of the absorption is discussed by studying the near-field distribution of modes 1 and 2 in the resonance peak, as shown in Figure 2a,b. The electric field distribution |*E*| is concentrated in the vacuum gap between the grating and the top of the structure. The presence of an electric field on the structure’s top indicates that the surface structure generates surface plasmon resonance (SPR). Figure 2c,d gives the normalized magnetic field |*H*| distributions corresponding to modes *f*_1_ and *f*_2_ in the cavity. At the VO_2_ and metal structure locations, the magnetic field exhibits transverse regions, which reflect SPR resonance characteristics. The magnetic field distribution |*H*| is also concentrated in the gap between the grating and the top of the structure, with longitudinal field regions within both modes, which exhibit Fabry–Perot-like gap plasmonic resonance (GPR) characteristics [10,30]. Therefore, the two-mode perfect absorption is observed from the field generated by the coupling of GPR and SPR resonance [31,32,33,34]. As the incident energy at the resonance frequency is strongly absorbed by the absorber with almost no energy reflection, resulting in near-perfect absorption.

**Figure 3:**
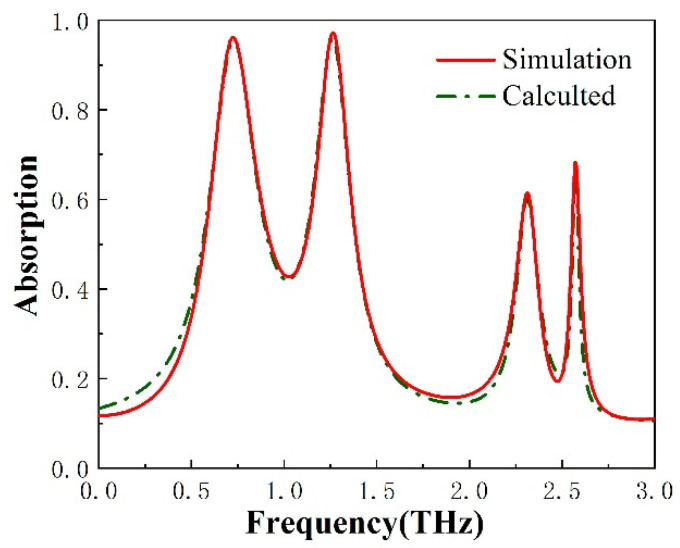
The simulated absorption spectrum and the calculated absorption spectrum of the functional device are given.

Furthermore, the absorption pattern of functional devices can be explained by the coupled-mode theory (CMT). CMT belongs to the parametric theoretical model, which can be used to reveal the physical principles of the coupling between artificial atoms in metamaterials. This algorithm has been tested and validated by many research components. According to CMT, the absorption intensity is given by [35]:

(3) $$A=\sum_{i=1}^{4}\left(\frac{4\gamma_{i}\delta_{i}}{{\left(\omega -\omega_{i}\right)}^{2}+{\left(\gamma_{i}+\delta_{i}\right)}^{2}}\right)$$

where *ω_i_* is the resonance frequency, and *γ_i_* and *δ_i_* are the time rate of the amplitude change and the dissipative losses in the guided resonance of the photonic crystal slab, respectively. As shown in Figure 3, The electromagnetic response obtained from the simulations and the CMT calculations are compared. The simulation results are in good agreement with the CMT calculated results in the operating frequency range of the absorption response.

**Figure 4:**
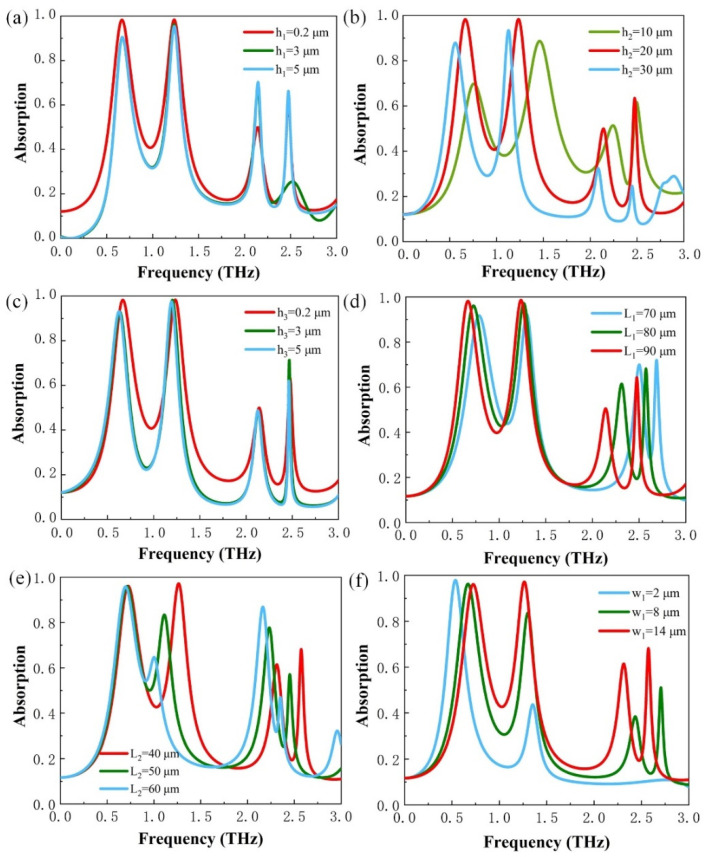
Absorption response of the metamaterial device in different (**a**) thickness of the VO_2_ backplane; (**b**) thickness of the substrate layer varies; (**c**) height of the VO_2_ and metal gratings vary; (**d**) and (**e**) grating width of the VO_2_ and metal varies; and (**f**) width of the gap between the gratings varies.

Through the detailed analysis above, it is believed that the device acts as a perfect absorber. To further demonstrate the perfect absorption induced by the device structure, we simulated the THz absorption spectra under different geometric parameters. We focus on variations of six parameters: the width of the two gratings and the gap between the gratings, the thickness of the VO_2_ backplane concerned, the thickness of the substrate, and the height of the gratings. As shown in Figure 4a, when the VO_2_ backplate is in the metallic state, changing the backplate thickness will affect the performance of the absorption peak and inhibit the absorption effect. It can be found that the best absorption effect is achieved at *h*_1_ = 0.2 μm. In contrast to the case of Figure 4a, as shown in Figure 4b, changing the polyimide spacer thickness not only affects the performance of the absorption peak but also the frequency of the absorption peak, which is red-shifted by increasing the thickness of the intermediate layer. The performance of the absorber can reach more than 98% of the two perfect absorption peaks at *h*_2_ = 20 μm. As shown in Figure 4c, there is an increase in absorbance at peak *f*_4_. The simulation results can demonstrate that the geometry parameters of the structure thickness have an important role in tuning the absorption performance of the metamaterial functional device.

As given in Figure 4d,e, the effect of the resonance peaks and the frequency of the absorber are greatly related to the grating width. The two parameters of the grating width *l*_1_ and *l*_2_, affect the absorption properties. When the value of the grating width *l*_1_ increases, the absorption peaks appear blue-shifted, absorption peaks *f*_1_ and *f*_2_ are suppressed, and absorption peaks *f*_3_ and *f*_4_ are enhanced. However, when the width of the grating *l*_2_ is increased, absorption peak *f*_1_ is almost constant, but the intensity of absorption peak *f*_2_ is significantly suppressed; the intensity of absorption peak *f*_3_ is significantly enhanced and the intensity of absorption peak *f*_4_ is suppressed. All other absorption peaks have a red-shifted except for the peak *f*_1_. As shown in Figure 4f, the width of the gap among the grids also has a significant effect on the absorption peaks. The three absorption peaks, except absorption peak *f*_1_, will appear blue-shifted and the absorption efficiency is also significantly suppressed with the decreasing width. The three absorption peaks, except for absorption peak *f*_1_, become red-shifted, while the absorption efficiency is enhanced with the increasing width. Through the analysis above, it can be explained that the adjustment of the geometrical parameters of the structure has an important influence on the absorption performance of the metamaterial device. Therefore, the appropriate adjustment of geometrical parameters can help the device in meeting the requirements of a wide range of applications.

**Figure 5:**
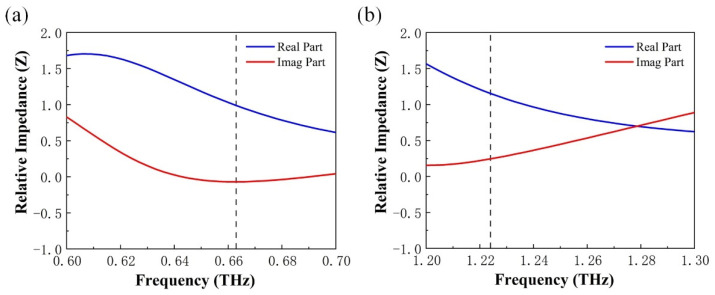
(**a**,**b**) the real and imaginary parts of the relative impedance of the perfect absorption peaks at 0.66 THz and 1.22 THz.

Impedance matching theory was used to gain an insight into the physics of the proposed device [36,37]. *S*-parameter retrieval gives the relative impedance as:

(4) $$Z=\pm \sqrt{\frac{{\left(1+S_{11}\right)}^{2}-S_{21}^{2}}{{\left(1-S_{11}\right)}^{2}-S_{21}^{2}}}$$

where *S*_11_ and *S*_22_ represent the reflection coefficient at port 1 and the transfer coefficient from port 1 to port 2, respectively. In this case, *Z* = *Z*_1_/*Z*_0_, where *Z*_1_ denotes the equivalent surface impedance of the device and *Z*_0_ = 377 W denotes the free space impedance. When *Z* = 1, the relative impedance of the device matches the free space impedance. This indicates that the device can achieve a perfect absorption response. The real part (Re(Z)) and the imaginary part (Im(Z)) of the impedance is a function of frequency when VO_2_ is in the metallic state. Figure 5a,b show the Re(Z)≈1,Im(Z)≈0 at the absorption peak (0.66 THz and 1.22 THz), respectively. This means that the impedance matching performance is perfect at this point [37,38].

**Figure 6:**
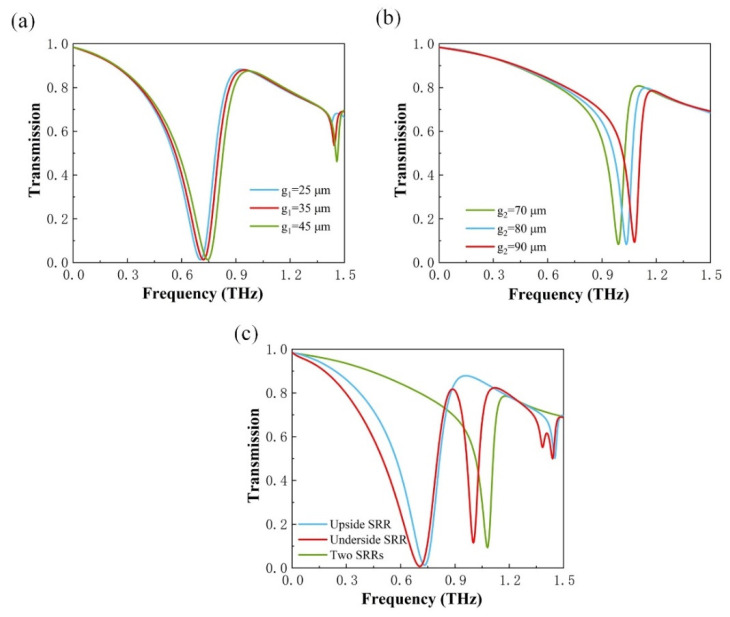
The effect of geometric parameters on resonance. (**a**) Adjustment of the size of *g*_1_ when the upside SRR is operating alone, (**b**) adjustment of the size of *g*_2_ when the underside SRR is operating alone. (**c**) Transmission amplitude when both SRRs are operating simultaneously.

Figure 6c shows that a transmission peak with a transmittance of more than 81% was obtained at 0.9 THz, which is expected because the insulating VO_2_ films are transparent to the THz wave. In addition, when VO_2_ is in the insulating state, resonance generated by the metal structure occurs. To further research these two kinds of resonances, the effect of structural geometries on the proposed metamaterial device’s transmission function is investigated. The variation of two different parameters is discussed: firstly, the size of the upside split-ring resonator (SRR) gap, and the second one is the underside SRR gap, with the SRR line width at 10 μm. As shown in Figure 6a, when the upside SRR is operating alone, adjusting the gap size has a minor effect on the amplitude of the resonances, but the change has a significant effect on the frequency of the resonances. With the increasing value of the gap size *g*_1_, the resonance’s frequency appears blue-shifted. As shown in Figure 6b, when the underside SRR has operated alone, changing the underside SRR gap size also has a slight effect on the amplitude of the resonances, but the change has a significant effect on the frequency of the resonances. The results show that the metamaterial resonance frequency has an obvious blue shift with an increase in gap size *g*_2_.

**Figure 7:**
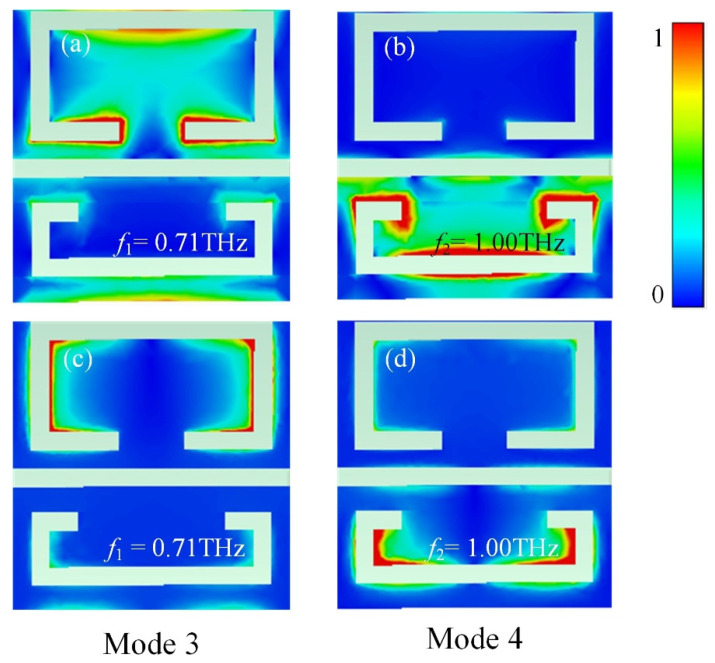
(**a**,**b**) The resonance electric field |*E*| and (**c**,**d**) the resonance magnetic field |*H*| for metamaterial devices.

Through the detailed analysis above, it can be believed that the size of the gap of the SRR is the key factor affecting the frequency of the resonances. To investigate the mode of operation of the transmittance, Figure 7a,b provide the electric field |*E*| distribution of the resonance at 0.71 THz and the resonance at 1.00 THz, and Figure 7c,d show the magnetic field |*H*| distribution. As shown in Figure 7a,c, the fields of the resonance are mostly concentrated on the bottom and two top corners of the upside SRR. As a result, the mode of operation at 0.71 THz should be caused by coupling between the dipole response and the LC resonance. At 1.00 THz, as shown in Figure 7b,d, the fields of resonance accumulate at the bottom and two top corners of the underside SRR, both the electric and magnetic field distributions are similar to the modes of the upside SRR. Therefore, we can conclude that the resonant modes of the resonances at 0.71 THz and 1.00 THz are the same.

**Figure 8:**
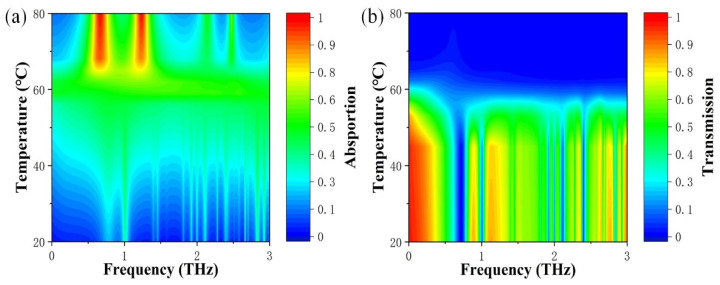
Calculated color maps for (**a**) absorbance and (**b**) transmittance spectra of metamaterial devices at different temperatures.

Based on the above analysis, a metamaterial device was designed and optimized with typical geometric parameters. To better demonstrate the superior and continuous tuning characteristics of the device, the color maps for transmission and absorption spectra of the device at different temperatures based on its material properties are shown in Figure 8a,b. The tunable function of the device can be achieved through the adjustment of temperature. When the temperature is 20 °C, VO_2_ is in the insulated state with the highest transmittance and the lowest corresponding absorbance. In this case, the device can be considered as an absorber in the off state and a transmissive device function with transmission peaks (>80%). When the temperature is 80 °C, VO_2_ is in the metallic state with the highest absorptance and the lowest corresponding transmittance. Interestingly, when the temperature increases, it exhibits a device with four absorption peaks, including two perfect absorption peaks with high absorbance (>98%), where the maximum tunable range of the absorption can be modulated from 3.9% to 98.3%, and the modulation amplitude of 94.2%. The transmission peak can be dynamically adjusted with temperature over a range of 0.02% to 81.8%, with a modification amplitude over 81%. Consequently, the state of the device can be switched between a tunable transmitter and a tunable absorber. In general, these results show that good performance can be achieved for both functions, which is beneficial in practical applications.

Finally, we provided a comparison of the device’s performance with others at THz frequencies. We listed the main properties of the different devices in Table 2. As a result, our proposed VO_2_-based THz metamaterial device not only has two switchable and tunable functions but also has a large increase in the modulation range.

## 4. Conclusions

In conclusion, the functional switchability and tunability of dual-functional THz metamaterial devices can be achieved by utilizing temperature driving phase change properties of the VO_2_ material. Through the simulation, the devices can be used in both tunable transmitters (transmission peak up to 81.8%) and tunable absorbers (absorption peak up to 98.3%). The mechanism of the devices is well illustrated by the CMT as well as the impedance matching theory. Therefore, a wide range of applications in the THz range, including switching, modulation, and sensing, can benefit from the development of these devices.

## Figures and Tables

**Table 1:** Parameters of material in simulation.

Material	Electric Conductivity	Thermal Conductivity	Epsilon	Tangent Delta
Gold	4.56 × 10^7^ S/m	314 W/K/m	/	/
Polyimide	/	0.2 W/K/m	3.5 + 0.00945i F/m	0.0027%

**Table 2:** Comparisons between THz metamaterial devices.

Sample Type	Number of Layers	Frequency (THz)	Modulation Type	Modulation Depth	Practical Implementation	Multifunction	Ref
VO_2_	2	0.1–1.0	Transmission	50%	device	no	[39]
W-doped VO_2_	2	0.3–2.3	Transmission	60%	device	no	[40]
VO_2_	3	3.7–9.7	Absorption	99.9%	model	no	[41]
VO_2_ nanowires	3	0.3–0.5	Polarization & Transmission	65%	device	yes	[42]
VO_2_	3	3.4–6.7	Reflection & Absorption	99.8%	model	yes	[43]
MoS_2_	3	0.6–1.2	Transmission	20%	device	no	[44]
Si_3_N_4_	4	0.2–0.7	transmission	25%	device	no	[45]
VO_2_	3	0.1–3.0	Transmission & Absorption	94.2%	model	yes	This work

## Data Availability

The data that support the plots within this paper are available from the corresponding authors on reasonable request.
